# Opportunity to Integrate the American Medical Association's Inclusive Language Guidance

**DOI:** 10.1089/heq.2023.0207

**Published:** 2024-03-13

**Authors:** Brelahn Wyatt-Nash, Ysehak Wondwossen, Monica A. Lutgendorf, Krista B. Highland

**Affiliations:** ^1^ Walter Reed National Military Medical Center, Bethesda, Maryland, USA.; ^2^ Eisenhower Army Medical Center, Fort Eisenhower, Georgia, USA.; ^3^ Department of Gynecologic Surgery and Obstetrics, Uniformed Services University, Bethesda, Maryland, USA.; ^4^ Department of Anesthesiology, Uniformed Services University, Bethesda, Maryland, USA.

**Keywords:** inclusive language, equity, justice, patient-centered care

## Abstract

Inclusive language is a cornerstone for inclusive, just, and equitable health care. While the American Medical Association released inclusive language guidance in 2021, it was unclear the extent to which physician practice organizations and their affiliated journals have adopted and promoted inclusive language. In our analysis, we found a lack of inclusive language resources across many physician practice organizations and their affiliated journals. Moreover, when guidance was provided by such entities, it was sometimes limited or not reflective of the American Medical Association recommendations. As such, many practice organizations and their journals have the opportunity to promote inclusive language.

## Inclusive Language: Importance and Impact

Inclusive language use is a way physicians may embody the American Medical Association (AMA) ethical principles of beneficence and nonmaleficence,^1^ as referring to people, their identities, bodies, and their care with dignity and respect is central to promoting their well-being and avoiding harming or injuring individuals. Unfortunately, noninclusive language is commonly present, embedded in the electronic health records fields (e.g., nonspecific “sex/gender” field in which assigned sex and gender are not disaggregated), diagnoses, and standard diagnostic classifications.^2^ The negative impacts of noninclusive language in medicine are significant, resulting in biases and stigma toward patients.^3–5^ Inclusive language goes beyond simple choice of words, as words build meaning within narratives and narratives can alter or reinforce beliefs and behaviors that compound inequities. Consequently, inclusive language is a cornerstone of delivering structurally competent^6^ and responsive care.

Inclusive language addresses many aspects of identity (e.g., race and ethnicity, sexual orientation, gender, disability), lived experiences (e.g., “person who is incarcerated” instead of “criminal”), and health care (e.g., “chest reconstruction” instead of “mastectomy” for trans men, trans masculine, and nonbinary people). Multiple publications encourage the use of person-first language when referring to people with health conditions and lived experiences. For example, instead of referring to someone as “an opioid addict,” a person-first approach would use “a person living with opioid-use disorder” instead.^7,8^ At times, inclusive language may also include identity-first language (e.g., “autistic people”), as members of communities have pride in and have reclaimed and destigmatized parts of their identities.^9,10^

Inclusive language includes the recognition of assigned sex and gender as distinct aspects of identity, that gender identity can be expansive and dynamic, and the agency each person has to self-identify their gender. For example, phrases such as “he/she” to refer to an unknown or unidentified person are noninclusive of people who use other pronouns. In contrast, “they” is an inclusive choice when referring to an unknown or unidentified person, and is grammatically sound.^11^ Inclusive language evolves over time, and hence, ensuring up-to-date knowledge is an important opportunity for physicians to ensure equitable health care practices.

Over the past few years, inclusive language guidelines have been released by major practice organizations and government entities such as the AMA,^12^ the American Psychological Association,^13^ and the Centers for Disease Control and Prevention.^14^ While these published guidelines have nuanced differences in specific terms, all describe recommendations regarding the use of person-first language and identity-first language (although consensus is not necessarily reached with all terms). In addition, these guidelines emphasize the importance of intentionality and awareness of how language shapes perception. For example, the AMA Advancing Health Equity: A Guide to Language, Narrative, and Concepts in 2021 was created in collaboration with the Association of American Medical Colleges,^12^ and delineates differences between the intent, impact, and the role of language in systemic health injustices in addition to specific language recommendations.^12^

Important goals of the AMA Guide include stimulating critical thinking about language, narrative, and concepts to work toward healthcare justice and health equity.^12^ While the AMA Guide was released more than 2 years ago, it is unclear the extent to which physician practice organizations and affiliated journals have adopted the recommendations of the AMA Guide. Therefore, the goal of this analysis was to evaluate the extent to which inclusive language recommendations in particular—or diversity, equity, inclusion, and justice (DEIJ) resources in general—are publicly accessible and published on physician practice organizations and their affiliated journal websites.

## Evaluating Accessible Information in Practice Organizations' and Journals' Websites

Practice organizations were identified through a search of the American Board of Medical Specialties (ABMS) website (www.abms.org). A total of 26 U.S. medical specialties and the AMA were identified (note: Psychiatry and Neurology are organized under one ABMS Board, but were disaggregated for this study), and their corresponding professional organization website was identified using the Google Search Engine (Mountain View, CA).

We searched practice organization and journal websites through December 16, 2023, using embedded search functions embedded within each practice organization's website for the following phrases: “inclusive language,” “inclusive terminology,” “diversity, equity, inclusion, justice (DEIJ) resources,” “DEI resources,” “diversity resources,” and “diversity.” If the embedded search function was not available or did not produce results, we used Google to search for these terms with the practice organization's name added to the search phrase. If there were multiple resources for a particular practice organization, results were prioritized as follows: (1) inclusive language guidance and resources, followed by (2) DEIJ resources in general; then if neither (1) nor (2) was identified, (3) public statements or policies regarding DEIJ commitment were identified. Published articles identified in the organization website searches were not extracted, as the goal was to identify public-facing, accessible guidance produced by the organizations.

Identified resources for each practice organization were searched as noted above, and data were extracted by one of the team members (K.B.H. and Y.W.); the data were then independently verified by at least one other team member (K.B.H., Y.W., and M.A.L.). Search information is collated into Table 1 in which each organization's website was identified as having accessible inclusive language guidance (Yes, No—but includes DEIJ resources and/or statement, and None).

**Table 1.** Practice Organizations, Flagship Journals, and Identified Guidance in Inclusive Language

Specialty, practice organization, and journal	Guidance or statement	Journal author guidance	Website includes accessible inclusive language guidance?^a,b^
American Medical Association *Journal of the American Medical Association*	Equity and the JAMA Network Inclusive Language for Reporting Demographic and Clinical Characteristics	February 2020: AMA Manual of Style (v11) (*Chapter 11.0 Correct and Preferred Usage),* “Guidance is also provided on inclusive language (e.g., sex/gender, presenting data in tables, personal pronouns, sexual orientation, race and ethnicity, age, socioeconomic status, and terminology for persons with diseases, disorders, or disabilities).”	Organization: Yes Journal: Yes
Allergy and Immunology American Academy of Allergy, Asthma & Immunology *The Journal of Allergy and Clinical Immunology*	AAAAI DIversity Equity and Inclusion Statement Statement Diversity, Equity, and Inclusion Initiatives Includes the journal's diversity statement that is in line with the AAAAI DEI statement. Link to DEI collection of articles published within journal (e.g., resources)	Author Guidelines: includes statement on inclusive language “Inclusive language acknowledges diversity, conveys respect to all people, is sensitive to differences, and promotes equal opportunities. Content should make no assumptions about the beliefs or commitments of any reader; contain nothing which might imply that one individual is superior to another on the grounds of age, gender, race, ethnicity, culture, sexual orientation, disability or health condition; and use inclusive language throughout. Authors should ensure that writing is free from bias, stereotypes, slang, reference to dominant culture and/or cultural assumptions. We advise to seek gender neutrality by using plural nouns (“clinicians, patients/clients”) as default/wherever possible to avoid using “he, she,” or “he/she.” We recommend avoiding the use of descriptors that refer to personal attributes such as age, gender, race, ethnicity, culture, sexual orientation, disability or health condition unless they are relevant and valid. When coding terminology is used, we recommend to avoid offensive or exclusionary terms such as “master,” “slave,” “blacklist,” and “whitelist.” We suggest using alternatives that are more appropriate and (self-) explanatory such as “primary,” “secondary,” “blocklist,” and “allowlist.” These guidelines are meant as a point of reference to help identify appropriate language but are by no means exhaustive or definitive.”	Organization: No—but includes DEIJ resources and/or statement Journal: Yes
Anesthesiology American Society of Anesthesiologists *Anesthesiology*	ASA Medical Student Component DEI Key Terms Note, promoted for student audience, but applicable for all Links to other inclusive language resources Some terminology and definitions are not consistent with AMA or APA guidelines (e.g., the “A” in LGBTQIA+ refers to asexual people and allies, but the latter is not cited as part of LGBTQIA+ acronym)	Author Guidelines: No specific recommendations for inclusive language	Organization: Yes Journal: No
Colon and Rectal Surgery American Society of Colon and Rectal Surgeons *Diseases of the Colon & Rectum*	None found	Author Guidelines: No specific recommendations for inclusive language	Organization: None Journal: No
Dermatology American Academy of Dermatology *Journal of the American Academy of Dermatology*	Diversity and the Academy Link to organizational DEI statement of intent Links to other DEI initiatives promoted by the organization Links to DEI resources	Author Guidelines: includes statement on inclusive language “Inclusive language acknowledges diversity, conveys respect to all people, is sensitive to differences, and promotes equal opportunities. Content should make no assumptions about the beliefs or commitments of any reader; contain nothing which might imply that one individual is superior to another on the grounds of age, gender, race, ethnicity, culture, sexual orientation, disability or health condition; and use inclusive language throughout. Authors should ensure that writing is free from bias, stereotypes, slang, reference to dominant culture and/or cultural assumptions. Seek gender neutrality by using plural nouns (“clinicians, patients/clients”) as default/wherever possible to avoid using “he, she,” or “he/she.” Avoid the use of descriptors that refer to personal attributes such as age, gender, race, ethnicity, culture, sexual orientation, disability or health condition unless they are relevant and valid. Refer to the current AMA style guide on race and ethnicity for a complete discussion and examples. These guidelines are meant as a point of reference to help identify appropriate language but are by no means exhaustive or definitive. Please see the full AMA Manual of Style for more information.”	Organization: No—but includes DEIJ resources and/or statement Journal: Yes
Emergency Medicine American College of Emergency Physicians *Annals of Emergency Medicine*	Diversity, Inclusion, and Health Equity Section Links to resources, news, and meeting/organization information EM Organizations Issue Shared Diversity Statement Statement	Author Guidelines: “Policy: Sex and Gender Equity in Research Annals endorses the SAGER (Sex and Gender Equity in Research) guidelines (1,2) and accordingly asks authors to include sex and gender considerations where relevant. The term sex should be used when reporting biological factors and gender should be used when reporting gender identity or psychosocial/cultural factors, and authors should avoid confusing the terms. If a sex and/or gender analysis was not conducted and the reason not obvious (e.g., a study of prostate cancer), the rationale should be given in the discussion.”	Organization: No—but includes DEIJ resources and/or statement Journal: Yes
Family Medicine American Academy of Family Physicians *American Family Physician*	Center for Diversity and Health Equity Links to other resources	Author Guidelines: No specific recommendations for inclusive language	Organization: No—but includes DEIJ resources and/or statement Journal: No
Internal Medicine American College of Physicians *Annals of Internal Medicine*	DEI Includes general resources	Author Guidelines: “Annals maintain an in-house style manual that is heavily based on principles outlined in the AMA Manual of Style 11th edition. Authors should adopt bias-free and person-first language, as outlined by the American Psychological Association.”	Organization: No—but includes DEIJ resources and/or statement Journal: Yes
Medical Genetics and Genomics American College of Medical Genetics and Genomics *Genetics in Medicine*	ACMG Diversity Statement Statement Diversity, Equity, and Inclusion Definitions Defines DEI, but does not provide explicit inclusive language information Provides links to external resources/websites with inclusive language information	Author Guidelines: includes statement on inclusive language, in addition to prompts for the authors to consider *Guidance on Language and Reporting on Diversity, Disability, Race, Ethnicity, Sex and Gender* When writing your article please bear in mind the following: Is there a description in the methods section of race/ethnicity, sex/gender variables that were collected? Is the source of racial/ethnic, sex/gender classification reported? (e.g., database, electronic health record, survey) Is there a description of how participant race/ethnicity, sex/gender was assigned? (e.g., participant self-report, participant selection of predetermined categories, investigator or clinician assignment) Participant self-report or selection is more rigorous than investigator- or clinician-assigned. Is a scientifically justified reason for collection of race/ethnicity, sex/gender data provided? Is a scientifically justified reason for classification by sex vs. gender provided? Are race and/or ethnicity being used as a proxy for genetic ancestry? If so, this is inappropriate. Is sex being used as a proxy for gender or vice versa? In most cases this would be considered inappropriate. Are white populations or European ancestry groups being used as reference categories in analysis? If so, is there a scientific justification for this comparison? In studies of genetic contributions to health disparities, are social determinants of health, environmental exposures and other likely relevant variables included? If not, is the possible role of these nongenetic factors discussed as a limitation? Is the study providing findings from underrepresented populations? If not, is a rationale provided, and discussion of the limitations of the generalizability of the study? Are terms used that make assumptions about the gender of persons based on anatomy, physiology, or biology? For example, is “pregnant women” being used rather than “pregnant persons”? “Women” rather than “persons with uteruses”? Are study findings inclusive of LGBTQIA+ persons? If not, is a rationale provided, and discussion of the limitations of the generalizability of the study? Inclusive language acknowledges diversity, conveys respect to all people, is sensitive to differences, and promotes equal opportunities. Content should make no assumptions about the beliefs or commitments of any reader; contain nothing which might imply that one individual is superior to another on the grounds of age, gender, race, ethnicity, culture, sexual orientation, disability or health condition; and use inclusive language throughout. Authors should ensure that writing is free from bias, stereotypes, slang, reference to dominant culture and/or cultural assumptions. We advise to seek gender neutrality by using plural nouns (“clinicians, patients/clients”) as default/wherever possible to avoid using “he, she,” or “he/she.” We recommend avoiding the use of descriptors that refer to personal attributes such as age, gender, race, ethnicity, culture, sexual orientation, disability or health condition unless they are relevant and valid. These guidelines are meant as a point of reference to help identify appropriate language but are by no means exhaustive or definitive. *Race, Ethnicity, and Genetic Ancestry* For race, ethnicity, and genetic ancestry terms please refer to “Updated Guidance on the Reporting of Race and Ethnicity in Medical and Science Journals published in JAMA” and follow the copy editing guidelines in the AMA Style guide.”	Organization: No—but includes DEIJ resources and/or statement Journal: Yes
Neurological Surgery American Association of Neurological Surgeons *Journal of Neurosurgery*	Statement by Black Neurosurgeons (June 2020) Statement Note: another webpage for diversity contains profiles of Diversity Committee members, but no resources for organization members	Author Guidelines: No specific recommendations for inclusive language	Organization: No–but includes DEIJ resources and/or statement Journal: No
Neurology American Academy of Neurology *Neurology*	Guiding Policy on Equity, Diversity, and Inclusion DEI at the AAN DEI resources linked include organization's DEI activities/programs (e.g., leadership and health care improvement programs, grants, scholarship opportunities), educational programs, resources, and statements/policies.	Author Guidelines: The Neurology journals follow the latest version of the AMA Manual of Style and the “ICMJE Recommendations for the Conduct, Reporting, Editing, and Publication of Scholarly work in Medical Journals.”	Organization: No—but includes DEIJ resources and/or statement Journal: No
Nuclear Medicine American College of Nuclear Medicine *Clinical Nuclear Medicine*	None Found	Author Guidelines: No specific recommendations for inclusive language	Organization: None Journal: No
Obstetrics and Gynecology American College of Obstetrics and Gynecology *Obstetrics and Gynecology*	Inclusive Language—Statement of Policy Policy addresses gender-related inclusive language, but no other areas of language Diversity, Equity, and Inclusive Excellence at ACOG Provides links to ACOG strategy, education and training, and clinical guidance and policy statements/.	Author Guidelines—Inclusive Language: includes statement on inclusive language found “The journal follows ACOG's Statement of Policy on Inclusive Language (https://www.acog.org/clinical-information/policy-and-position-statements/statements-of-policy/2022/inclusive-language). Please be mindful of using gendered descriptors in your article. Instead of “women” and “females,” consider using the following: “Individuals” “Patients” “Participants” “People” (not “persons”) “Women and transgender men” “Women and gender-expansive patients” “Women and all those seeking gynecologic care” In secondary analyses or database studies, please retain the language used by parent study or database source for consistency.”	Organization: Yes Journal: Yes
Ophthalmology American Academy of Ophthalmology *Ophthalmology*	Diversity, Equity, and Inclusion Includes links for policies, initiatives, programs, resources, and activities	Author Guidelines: includes statement on inclusive language *“*Use of inclusive language Inclusive language acknowledges diversity, conveys respect to all people, is sensitive to differences, and promotes equal opportunities. Content should make no assumptions about the beliefs or commitments of any reader; contain nothing which might imply that one individual is superior to another on the grounds of age, gender, race, ethnicity, culture, sexual orientation, disability or health condition; and use inclusive language throughout. Authors should ensure that writing is free from bias, stereotypes, slang, reference to dominant culture and/or cultural assumptions. We advise to seek gender neutrality by using plural nouns (“clinicians, patients/clients”) as default/wherever possible to avoid using “he, she,” or “he/she.” We recommend avoiding the use of descriptors that refer to personal attributes such as age, gender, race, ethnicity, culture, sexual orientation, disability or health condition unless they are relevant and valid. When coding terminology is used, we recommend to avoid offensive or exclusionary terms such as “master,” “slave,” “blacklist,” and “whitelist,” We suggest using alternatives that are more appropriate and (self-) explanatory such as “primary,” “secondary,” “blocklist,” and “allowlist,” These guidelines are meant as a point of reference to help identify appropriate language but are by no means exhaustive or definitive.”	Organization: No—but includes DEIJ resources and/or statement Journal: Yes
Orthopedic Surgery American Academy of Orthopedic Surgeons *Journal of the AAOS*	Diversity & AAOS Provides statements and reports/activities, but does not include accessible DEI education resources	Author Guidelines: No specific recommendations for inclusive language	Organization: No—but includes DEIJ resources and/or statement Journal: No
Otolaryngology—Head and Neck Surgery American Academy of Otolaryngology—Head and Neck Surgery *Otolaryngology—Head and Neck Surgery*	Diversity, Equity, and Inclusion Statement from the organization on DEI and grants Implicit Bias Series Educational DEI resources Race and Ethnicity: Guidance on Publishing/Reviewing Studies and Diversity Initiatives Recorded webinar from AMA and JAMA regarding inclusive language in race and ethnicity	Author Guidelines: No specific recommendations for inclusive language, but provided information regarding inclusion of author pronouns: “Authors may now include their personal pronouns in the author bylines of their published articles and on Wiley Online Library. Authors will never be required to include their pronouns; it will always be optional for the author. Authors can include their pronouns in their manuscript upon submission and can add, edit, or remove their pronouns at any stage upon request. Submitting/corresponding authors should never add, edit, or remove a coauthor's pronouns without that coauthor's consent. Where post-publication changes to pronouns are required, these can be made without a correction notice to the paper, following Wiley's Name Change Policy to protect the author's privacy. Terms which fall outside of the scope of personal pronouns (e.g., proper or improper nouns), are currently not supported.”	Organization: No—but includes DEIJ resources and/or statement Journal: No
Pathology College of American Pathologists *Archives of Pathology & Laboratory Medicine*	Public policy regarding DEI This document is not accessible to public and therefore, it is unknown what is in it, but the assumption is it is a policy. Advocating for Health Equity Indicates policies the organization supports to improve health equity	Author Guidelines: Refers to AMA Manual of Style “Manuscripts should be prepared in accordance with the American Medical Association (AMA) Manual of Style, 10th edition.”	Organization: No—but includes DEIJ resources and/or statement Journal: No
Pediatrics American Academy of Pediatrics *Pediatrics*	Words Matter: AAP Guidance on Inclusive, Anti-biased Language Provides an overview of inclusive language terminology across areas of identity and experiences Includes some terminology that is not consistent with the AMA or APA guidelines (e.g., using a “/” between race and ethnicity, as well as gender and sexual orientation)	Author Guidelines: includes statement on inclusive language; provides reference link to AMA Manual of Style (v11, Chapter 11) Includes a table with preferred terms and recommendations for inclusive images. “Use of Inclusive Language Articles published in Pediatrics should use the most inclusive language possible. These recommendations are intended to guide authors but are not comprehensive. As the preferred terminology related to inclusive language evolves over time, these recommendations will be updated continuously. Please reach out to the editorial office for clarifications or suggestions. Inclusive Language Person-first language, which emphasizes the individual or group rather than the condition, disease, or situation, should generally be used, e.g., “child(ren) with diabetes” and “child(ren) with obesity” rather than “diabetic child(ren)” and “obese child(ren).” Exceptions to first-person language include certain identity-first language for individuals and groups who prefer it, e.g., “Deaf child(ren)” or “autistic child(ren).” Race and ethnicity categories should be capitalized, including the White race. Race and ethnicity should be used as adjectives rather than nouns, e.g., “Hispanic individuals” rather than “Hispanics.” Race and ethnicity should be treated as separate categories rather than merging them, e.g., “race and ethnicity” rather than “race/ethnicity.” Articles that report race and/or ethnicity should use the specific terms used in data collection or in the original study referenced. The terms should be accurate, understandable to study participants, and consistent with participants' self-understanding. Refer to gender identity using terms such as “cisgender or transgender,” “man or woman,” “gender-nonbinary,” “genderqueer,” or “agender person,” rather than “transgendered,” “transsexual,” or “transvestite.” Refer to the community as “transgender and non-binary” or “gender diverse,” rather than “gender non-conforming.” Refer to sexual orientation using specific terms such as “heterosexual,” “lesbian,” “gay,” “bisexual,” “queer,” rather than terms such as “homosexual” or “non-heterosexual.” Refer to the “LGBTQ+ community” rather than the “gay community” unless referencing specific subgroups. Restrict the use of “men who have sex with men” to refer to behaviors rather than to sexual orientation. Both pregnant women” and “pregnant people” are acceptable terms. Avoid substituting “pregnant women” with phrases such as “birthing people” or “people with uteruses.” Neutral terms, such as “pregnant patients” and “pregnant people” are inclusive alternatives. Authors of research studies should use the specific terms used in data collection or in the original study referenced.”	Organization: Yes Journal: Yes
Physical Medicine and Rehabilitation (PM&R) American Academy of PM&R *PM&R: The Journal of Injury, Function, and Rehabilitation*	Advancing DIversity and Inclusion Information regarding the organization's efforts and strategic plans for improving DEI; contains a link to additional resources (presentations)	Author Guidelines: “Manuscripts should be prepared in accordance with the AMA Manual of Style, 10th edition, and/or the ICMJE Recommendations for the Conduct, Reporting, Editing, and Publication of Scholarly Work in Medical Journals.”	Organization: No—but includes DEIJ resources and/or statement Journal: No
Plastic Surgery American Society of Plastic Surgeons *Plastic and Reconstructive Surgery*	Diversity & Inclusion Resources Includes mission statement and goals of DEI committee, as well as links to resources (e.g., recommended readings)	Author Guidelines: No specific recommendations for inclusive language	Organization: No—but includes DEIJ resources and/or statement Journal: No
Preventive Medicine American College of Preventive Medicine *American Journal of Preventive Medicine*	Policy Statement on Health Equity Statement	Author Guidelines: provides guidance for sex and gender reporting; links to the Sex and Gender Equity (SAGER) Guidelines (which include outdated and noninclusive terminology) and Elsevier Diversity, Equity and Inclusion in Publishing sites. “In general, AJPM follows the American Medical Association Manual of Style, 10th edition: http://www.amamanualofstyle.com/. Please refer to this manual if you have questions about formatting or structure that are not covered in this document.” “Reporting sex- and gender-based analyses Reporting guidance For research involving or pertaining to humans, animals or eukaryotic cells, investigators should integrate sex and gender based analyses into their research design according to funder/sponsor requirements and best practices within a field. Authors should address the sex and/or gender dimensions of their research in their article. In cases where they cannot, they should discuss this as a limitation to their research's generalizability. Importantly, authors should explicitly State what definitions of sex and/or gender they are applying to enhance the precision, rigor and reproducibility of their research and to avoid ambiguity or conflation of terms and the constructs to which they refer (see Definitions section below). Authors can refer to the Sex and Gender Equity in Research (SAGER) guidelines and the SAGER guidelines checklist. These offer systematic approaches to the use and editorial review of sex and gender information in study design, data analysis, outcome reporting and research interpretation—however, please note there is no single, universally agreed-upon set of guidelines for defining sex and gender. Definitions Sex generally refers to a set of biological attributes that are associated with physical and physiological features (e.g., chromosomal genotype, hormonal levels, internal and external anatomy). A binary sex categorization (male/female) is usually designated at birth (“sex assigned at birth”), most often based solely on the visible external anatomy of a newborn. Gender generally refers to socially constructed roles, behaviors, and identities of women, men and gender-diverse people that occur in a historical and cultural context and may vary across societies and over time. Gender influences how people view themselves and each other, how they behave and interact and how power is distributed in society. Sex and gender are often incorrectly portrayed as binary (female/male or woman/man) and unchanging whereas these constructs actually exist along a spectrum and include additional sex categorizations and gender identities such as people who are intersex/have differences of sex development or identify as non-binary. Moreover, the terms “sex” and “gender” can be ambiguous—thus it is important for authors to define the manner in which they are used. In addition to this definition guidance and the SAGER guidelines, the resources on this page offer further insight around sex and gender in research studies.”	Organization: No—but includes DEIJ resources and/or statement Journal: Yes
Psychiatry American Psychiatric Association *The American Journal of Psychiatry*	Definitions of Gender, Sex, and Sexual Orientation and Pronoun Usage Brief explanation on assigned sex, gender, and sexual orientation with a list of pronouns lack of further inclusive terminology outside of these topic areas Mental Health Language Guide Links to guide on inclusive language related to mental health and health care	Author Guidelines: “All primary research reports are required to include information on the age, sex, gender identity, race, and ethnicity of the study subjects. The Methods section should include an explanation of how each participant characteristic was identified (e.g., self-report, investigator observed) and the source of the classifications used (e.g., U.S. Census data, PhenX toolkit). If the study design precluded the acquisition of any of these participant characteristics, an explanation should be provided. Studies that include predominantly White participants should acknowledge this limitation and note that findings may not generalize to non-White participants. Race and ethnicity should be treated as social constructs and when indicated, should be discussed in relation to the social, environmental, and economic factors in the study population. Description of subject characteristics should avoid the use of terminology that could stigmatize people with a psychiatric or addictive disorder. For all manuscripts reporting data derived from the study of human participants, authors are required to answer Yes/No to the following questions: Does your submission indicate how participant race and participant ethnicity were ascertained? Does your submission distinguish between assigned sex at birth and gender identity? Does your submission indicate how sex and gender were ascertained?”	Organization: Yes Journal: Yes
Radiology American College of Radiology *Journal of the American College of Radiology*	Diversity Glossary Provides information on some inclusive language, as well as resources for AMA guide	Author Guidelines: No specific recommendations for inclusive language	Organization: Yes Journal: No
Surgery American College of Surgeons *Journal of the American College of Surgeons*	ACS DEI and Antiracism Lexicon Provides information on terminology. Terminology and definitions are not always consistent with AMA and APA guidelines (although AMA guidelines are cited)	Author Guidelines: No specific recommendations for inclusive language	Organization: No—but includes DEIJ resources and/or statement Journal: No
Thoracic Surgery The American Association for Thoracic Surgery *The Journal of Thoracic and Cardiovascular Surgery*	Diversity, Inclusion, and Equity Statement Statement	Author Guidelines: No specific recommendations for inclusive language	Organization: No—but includes DEIJ resources and/or statement Journal: No
Urology American Urological Association *The Journal of Urology*	Diversity, Equity, and Inclusion Links to statement Links to DEI resources, programs	Author Guidelines: No specific recommendations for inclusive language	Organization: No—but includes DEIJ resources and/or statement Journal: No

^a^ Organization rating scale: Yes, No—but includes DEI resources and/or statement, None.

^b^ Journal rating scale: Yes or No.

AMA, American Medical Association; DEIJ, Diversity, Equity, Inclusion, and Justice.

Affiliated journals were identified for each of the 26 U.S. physician practice organizations. For each journal, we navigated to the author guidelines and instruction page and searched for the following phrases: “inclusive,” “sex,” “gender,” “race,” “ethnicity,” and “language.” Similar to organization's websites, each journal's author's instructions were identified as having inclusive language guidance (Yes or No). If yes, information regarding the specific inclusive language guidance was included in the summary table (Table 1).

Aside from the AMA, five practice organizations published explicit inclusive language guidance, although this guidance was not always consistent with the AMA (or APA) Guide. Half of the practice organizations (*n*=13) had listed resources and/or links to DEIJ resources that organization members and members of the public could access. Five practice organizations did not contain easily accessible DEIJ resources but included a public statement regarding the organization's commitment to DEIJ. Two practice organizations lacked accessible inclusive language guidance or DEIJ-related resources or statements. Of organization websites that contained accessible inclusive language guidance, the guidance varied, with some including limited scope (e.g., refer only to assigned sex and gender) or including information that was inconsistent with the AMA Guide.

For affiliated journal websites, 15 (57%) included explicit inclusive language guidance, however, the depth and scope of the guidance varied. For example, some journals included only recommendations regarding assigned sex and gender terminology, while others included more recommendations regarding reporting and terminology for assigned sex, gender, race, ethnicity, and disability. Four organization–journal combinations (15%) had accessible inclusive language guidance across both their organization and journal websites.

## Where to Go from Here

Lack of accessible and consistent inclusive language guidance and existing discrepancies between practice organizations and journal recommendations provide an opportunity for improvement to support and recommend inclusive language in articles. Given that the AMA Manual of Style updates guidance regularly (e.g., language regarding race and ethnicity reporting), with plans for ongoing updates,^15^ it will be important for practice organizations and journals to implement responsive inclusive language recommendations and DEIJ resources. Practice organizations are an important potential source of institutional and structural inequities, or they can serve as a proactive force to decrease inequities and bias at an institutional level.

While some practice organizations may encourage members to advocate for inclusive language changes (e.g., medical record field changes and updated electronic medical records), having accessible inclusive language guidance published and championed by individual practice organizations may accelerate ongoing institutional improvements and advocacy. Moreover, the enhanced accessibility of such inclusive language guidance will more consistently communicate the importance of integrating inclusive language throughout physicians' practices and work environments.

Many approaches could enhance organizational inclusive language implementation. Such efforts can increase the inclusivity and more accurately describe health and health care within a structurally responsive manner.^6^ Practice organizations could collectively agree to adopt, disseminate, and promote the AMA Guide in their organization-specific guidance. Accrediting bodies (such as the Joint Commission, Association of American Medical Colleges) and publishers could bolster inclusive language guidance and consistently refer to the AMA Guide. Increased transparency of inclusive language development and inclusion of diverse representation in the process are critical to elevate the voices and lived experiences of diverse individuals *and* ensure contributors are provided public recognition. Lastly, the AMA could ensure that inclusive language recommendations in its Manual of Style *and* the Guide are sufficiently flexible with regular updates to reflect the evolution of inclusive language.

## Limitations to the Present Evaluation

Our sampling included current ABMS specialties, however, this does not encompass all medical specialties. Our methodology may have missed additional organizations and subspecialty organizations within the specialties queried with their own inclusive language guidelines and journal recommendations. In addition, statements were located by searching public facing websites and journal submission portals, which may have missed nonpublicly available guidelines.

## Abbreviations Used

ABMS: American Board of Medical Specialties
AMA: American Medical Association
DEIJ: diversity, equity, inclusion, and justice
SAGER: Sex and Gender Equity in Research
